# The associations among iron metabolism markers, iron supplementation regimen, and sepsis-induced myocardial injury: a retrospective study

**DOI:** 10.3389/fnut.2026.1858343

**Published:** 2026-07-15

**Authors:** Xijia Wang, Xiaoying Wang, Yanling Liu, Jinming Ma, Silong Wu

**Affiliations:** 1 Department of Cardiovascular, The First Affiliated Hospital of Zhengzhou University, Zhengzhou, China; 2 Department of Rheumatology and Immunology, The First Affiliated Hospital of Zhengzhou University, Zhengzhou, China; 3 Department of Laboratory Medicine, The Third Affiliated Hospital of Sun Yat-sen University, Guangzhou, China; 4 Department of Burns, Nanfang Hospital of Southern Medical University, Guangzhou, China

**Keywords:** iron metabolism markers, iron supplementation regimen, MIMIC database, sepsis, sepsis-induced myocardial injury

## Abstract

**Background:**

Sepsis is a life-threatening organ dysfunction caused by a dysregulated host response, among which sepsis-induced myocardial injury (SIMI) is a common and severe complication. Iron metabolic disorders are closely associated with infection, inflammation, oxidative stress, and organ damage. However, their relationship with the prognosis of sepsis and SIMI, as well as the safety and benefits of iron supplementation therapy, remain unclear. This study aimed to investigate these associations.

**Methods:**

Based on the MIMIC-IV database, a retrospective cohort analysis was conducted in adult patients with sepsis admitted to the intensive care unit (ICU). Iron metabolism biomarkers and clinical data were extracted. Analytical methods included Cox regression, restricted cubic splines (RCS), and propensity score matching (PSM).

**Results:**

A total of 3,360 patients were enrolled. Hyperferritinemia was associated with an increased 30-day mortality (hazard ratio (HR) = 1.56, 95% confidence interval (CI): 1.195–2.038, *p* < 0.001). In contrast, higher transferrin levels (HR = 0.552, *p* < 0.001) and higher total iron-binding capacity (TIBC) (HR = 0.559, *p* < 0.001) exhibited protective effects. Serum iron levels did not correlate well with mortality. RCS analysis untangled a nonlinear relationship between iron metabolism markers and sepsis, whereas a linear association was observed between ferritin and SIMI. After propensity score matching (PSM), intravenous iron supplementation was associated with a lower 30-day mortality rate (10.16% vs. 27.81%, HR = 0.251, *p* < 0.001).

**Conclusion:**

Ferritin, transferrin, and total iron-binding capacity (TIBC) are of significant value for risk stratification in sepsis. Intravenous iron supplementation may improve short-term prognosis without increasing the risk of subsequent SIMI; however, this conclusion requires validation through prospective studies.

## 1 Introduction

Sepsis, triggered by acute infection and characterized by dysregulated host inflammatory responses and multiple organ dysfunction, is one of the leading causes of critical illness. The incidence of sepsis-induced myocardial injury (SIMI) ranges from 18 to 60% (1). A prospective cohort study demonstrated that the in-hospital mortality rate among patients with SIMI was 35% (2). As numerous studies have revealed a close association between the onset and progression of sepsis and novel forms of cell death such as ferroptosis and cuproptosis, increasing attention has been directed toward dysregulated trace element metabolism (3).

Iron is an indispensable trace element in the human body, participating in multiple fundamental physiological processes essential for sustaining life activities, including DNA synthesis, cellular energy metabolism, hemoglobin-mediated oxygen transport, ATP production, and the maintenance of immune function. The biological functions of iron primarily rely on its ability to reversibly gain or lose a single electron, thereby engaging in redox reactions. However, this inherent property also renders iron a potent catalyst for the generation of reactive oxygen intermediates (ROIs), exerting dual effects on cellular homeostasis maintenance and pathological damage (4). By serving as the core component of heme groups, iron–sulfur (Fe–S) clusters, ferritin, and other specialized functional groups, iron is integrated into proteins to perform critical physiological roles and regulate their reactivity (5).

Beyond its crucial role in host physiological processes, iron is also vital for the survival, proliferation, and pathogenicity of most pathogenic microorganisms. The virulence of the vast majority of pathogenic bacteria is highly dependent on available iron sources. Clinically common pathogens such as *Escherichia coli* and *Klebsiella pneumoniae* have evolved sophisticated adaptive mechanisms to scavenge and chelate iron from host iron-binding proteins like transferrin, supporting their own replication and invasion (6). This competition for iron between the host and pathogens has given rise to the concept of “nutritional immunity,” an important host defense strategy characterized by a rapid reduction in circulating serum iron levels while sequestering substantial amounts of iron within intracellular compartments. This process limits the bioavailable iron accessible to pathogens and inhibits microbial proliferation (7). However, this defensive mechanism carries potential pathological risks: the excessive accumulation of “free” labile iron in the cytoplasm can trigger lipid peroxidation, induce ferroptosis (8), and ultimately lead to multi-organ dysfunction (9, 10). On the other hand, intracellular iron accumulation resulting from high levels of hepcidin leads to “functional iron deficiency,” which is a significant cause of anemia (11). Sepsis-associated anemia has been confirmed by multiple studies to be closely linked to poor patient prognoses, prolonged hospital stays, and increased mortality (12).

The safety and efficacy of iron supplementation during sepsis remain controversial, with primary concerns being that iron may induce immune dysfunction, thereby promoting infection and multi-organ injury (13–16). This study aimed to investigate the relationship between iron metabolism markers, iron supplementation, and 30-day outcomes as well as the occurrence of myocardial injury in patients with sepsis.

## 2 Methods

### 2.1 Population and dataset

This retrospective observational cohort study utilized data from the Medical Information Mart for Intensive Care IV (MIMIC-IV-3.1, https://physionet.org/content/mimiciv/) (17), which contains records of intensive care unit (ICU) admissions at Beth Israel Deaconess Medical Center in Boston, Massachusetts, from 2008 to 2019. Access to the database was granted upon completion of the required training (certification number: 73808475). Given the use of de-identified retrospective data, the requirement for individual informed consent was waived by the Institutional Review Boards of Beth Israel Deaconess Medical Center and the Massachusetts Institute of Technology.

**Figure 1:**
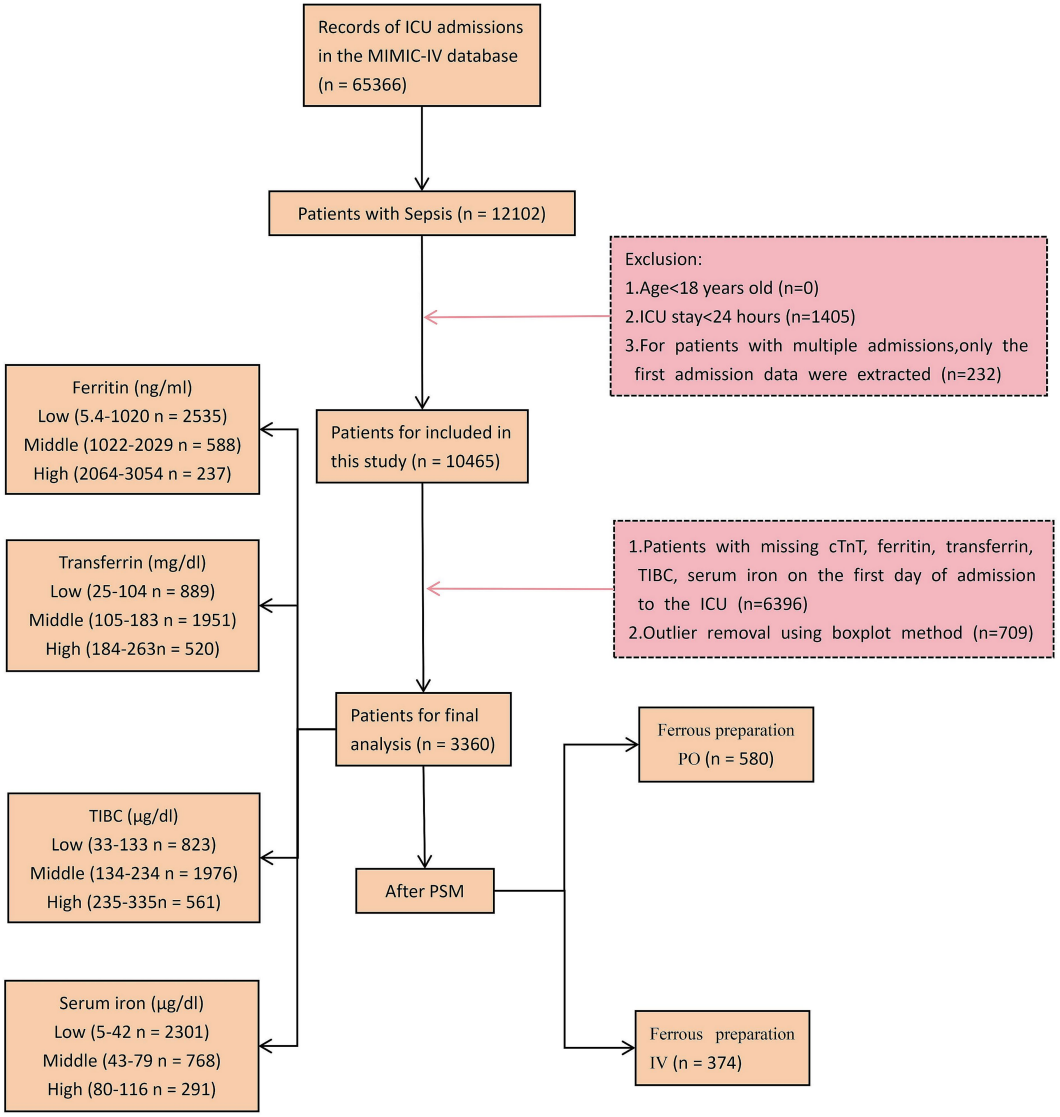
Flow chart of the study design. TIBC, total iron-binding capacity; PSM, propensity score matching; cTnT, cardiac troponin T; PO, per os; IV, intravenous.

Inclusion criteria were as follows: (1) age ≥18 years; (2) fulfillment of the diagnostic criteria for Sepsis 3.0 (18); and (3) ICU length of stay exceeding 24 h. Exclusion criteria included: (1) pre-existing cardiopulmonary diseases present prior to ICU admission that could elevate cardiac troponin T (cTnT) levels; (2) missing data for key variables; and (3) only the first ICU admission was analyzed. Diagnoses were identified through manual review of the International Classification of Diseases, Ninth Revision (ICD-9) and Tenth Revision (ICD-10) diagnosis codes (specific codes for all relevant diseases are detailed in Supplementary Table S1). The study flow chart is presented in Figure 1. Extracted data included demographic information, vital signs, clinical management measures, comorbidities, disease severity scores at admission, and laboratory parameters, as outlined in Table 1.

**Table 1:** Summary of extracted baseline data.

Pre-existing cardiopulmonary diseases	ACS, cardiomyopathy, myocarditis, endocarditis, COPD, CHF, cardiac arrest, AF, VF, VFL
Demographic data	Age, sex, race
Vital signs	HR, RR, SpO_2_, Nbpm, T
Clinical management measures	Mechanical ventilation, CRRT, epinephrine, norepinephrine, ferrous preparation (PO/IV)
Comorbidities	AKI, CKD, HLD, HTN, T2DM
Laboratory parameters	RBC, WBC, RDW, PLT, Hb, HCT, TB, ALT, Glu, Alb, AST, PT, INR, APTT, Scr, BUN, AG, PO_2_, PCO_2_, pH, K^+^, Na^+^, Ca^2+^, Cl^−^, cTnT, SI, SF, TF, TIBC, LDH, Lac
Disease severity scores at admission	SOFA, APS III, SIRS, SAPS II, OASIS, GCS, Charlson

HR, heart rate; Nbpm, non-invasive blood pressure mean; RR, respiratory rate; SpO_2_, oxygen saturation; T, body temperature; CRRT, renal replacement therapy; PO, per os; IV, intravenous; AKI, acute kidney injury; CKD, chronic kidney disease; HLD, hyperlipidemia; HTN, hypertension; T2DM, type 2 diabetes mellitus; RBC, red blood cell count; WBC, white blood cell count; RDW, red cell distribution width; PLT, platelet count; Hb, hemoglobin; HCT, hematocrit; TB, total bilirubin; ALT, alanine aminotransferase; Glu, serum glucose; Alb, albumin; AST, aspartate aminotransferase; PT, prothrombin time; INR, international normalized ratio; APTT, activated partial thromboplastin time; Scr, serum creatinine; BUN, blood urea nitrogen; AG, anion gap; PO_2_, partial pressure of oxygen; PCO_2_, partial pressure of carbon dioxide; K^+^, serum potassium; Na^+^, serum sodium; Ca^2+^, serum calcium; Cl^−^, serum chloride; cTnT, cardiac troponin t; SI, serum iron; SF, ferritin; TF, transferrin; TIBC, total iron-binding capacity; LDH, lactate dehydrogenase; Lac, lactate; SOFA, Sequential Organ Failure Assessment; APS III, Acute Physiology Score III; SIRS, Systemic Inflammatory Response Syndrome; SAPS II, Simplified Acute Physiology Score II; OASIS, Oxford Acute Severity of Illness Score; GCS, Glasgow Coma Scale; Charlson, Charlson Comorbidity Index; ACS, acute coronary syndrome; COPD, chronic obstructive pulmonary disease; CHF, chronic heart failure; AF, atrial fibrillation; VF, ventricular fibrillation; VFL, ventricular flutter.

### 2.2 Exposure and outcomes of this study

The primary outcome was 30-day all-cause mortality, and the secondary outcome was the occurrence of SIMI. The 99th percentile upper reference limit for cTnT in our center was 0.01 ng/mL. SIMI was defined as a cTnT level exceeding 0.01 ng/mL (19, 20). The study evaluated patients who had received iron therapy prior to admission to the ICU. Patients whose first cTnT measurement upon ICU admission exceeded 0.01 ng/mL were considered to have developed SIMI. Data on iron supplement use were derived from prescription records, but data on specific dosages were not obtained.

### 2.3 Statistical analysis

Patient baseline characteristics are summarized as follows: continuous variables are presented as medians with interquartile ranges, and categorical variables are expressed as frequencies with percentages. Intergroup differences were assessed using the Kruskal-Wallis test for continuous variables and the chi-square test for categorical variables. Outliers among the four iron metabolism biomarkers were excluded using the boxplot method. This approach is based on the data quantiles and the interquartile range (IQR). Data points falling outside the interval defined by Q1–1.5 × IQR to Q3 + 1.5 × IQR were identified as outliers. Subsequently, the four iron metabolism markers were categorized into low, medium, and high groups using equal-width binning. Samples with more than 25% missing values for any variable were excluded, and the remaining missing data were imputed using multiple imputation.

Kaplan–Meier survival curves were constructed to visualize survival probabilities across groups, and the log-rank test was employed to compare survival distributions between groups. A multivariable Cox proportional hazards model was used to evaluate 30-day mortality.

To assess the association between different groups and 30-day mortality, three sequentially adjusted models were developed: Model 1 was unadjusted; Model 2 was adjusted for baseline demographics and vital signs; and Model 3 was further adjusted for key laboratory indicators, complications, and disease severity scores.

A restricted cubic spline (RCS) model was employed to investigate the dose–response relationship between four iron metabolism indicators and the risks of 30-day all-cause mortality and sepsis-induced myocardial injury. For variables exhibiting a nonlinear dose–response relationship, a segmented threshold effect analysis was subsequently conducted to identify inflection points and their critical values. The hazard ratio (HR) was calculated separately for the segments before and after each identified inflection point.

To minimize confounding bias, a propensity score approach was adopted. A 1:1 propensity score matching (PSM) algorithm was applied to compare patients subjected to oral iron supplementation versus those receiving intravenous iron supplementation, thereby ensuring a balanced distribution of baseline characteristics. Adequacy of matching was confirmed using a standardized mean difference (SMD) threshold of less than 0.1.

Furthermore, the primary outcome (30-day mortality) was further examined in pre-specified subgroups to assess the potential modifying effects of key patient characteristics on the observed associations. Stratification was performed based on age, sex, race, comorbidities (anemia, HTN, AKI, CKD, T2DM, and HLD), baseline treatments (CRRT, mechanical ventilation), and baseline medications (epinephrine, norepinephrine). The significance of interaction effects was determined by the *p*-value for interaction.

Data extraction was performed using PostgreSQL (v13.7.1) and Navicat Premium (version 15) software by executing structured query language (SQL) queries. All statistical analyses were conducted using R software (version 4.2.3; R Foundation, http://www.R-project.org) and Free Statistics software (version 2.0). A two-sided *p-*value of less than 0.05 was considered to indicate statistical significance.

## 3 Results

### 3.1 Baseline characteristics of patients with sepsis

A total of 3,360 patients with severe sepsis were enrolled in this study. Among them, 784 patients died within 30 days, and 632 patients exhibited SIMI. Baseline characteristics of the patients are presented in Table 2. Notably, patients who died within 30 days or developed SIMI had significantly elevated ferritin levels, whereas their total iron-binding capacity (TIBC) and transferrin levels were significantly decreased (detailed data are shown in Supplementary Table S2). Among the 30-day non-survivors, higher cTnT levels were observed, along with a greater proportion of patients with SIMI, indicating that SIMI is associated with poor prognosis.

**Table 2:** Baseline characteristics associated with 30-day mortality in patients.

Variables	Overall	Survival	Death	*P*
*N*	3,360	2,576	784	
*Demographic variables*				
Age years	74 (63–82.25)	72 (62–81)	77 (67–85)	<0.001
Gender (%)				
Female	1,394 (41.49)	1,068 (41.46)	326 (41.58)	0.985
Male	1966 (58.51)	1,508 (58.54)	458 (58.42)	
Race (%)				
White	2,159 (64.26)	1,680 (65.22)	479 (61.10)	<0.001
Black	391 (11.64)	329 (12.77)	62 (7.91)	
Other	810 (24.11)	567 (22.01)	243 (30.99)	
Weight lbs	77.9 (64.2–94.5)	78 (64.262–95.075)	77 (64–93.5)	0.220
*Vital signs*				
HR bpm	94 (80–110)	94 (80–110)	94 (81–110)	0.876
RR insp/min	21 (17–25)	21 (17–25)	21 (18–26)	0.048
T °F	98.3 (97.6–98.9)	98.3 (97.7–99)	98.1 (97.6–98.8)	0.336
SpO_2_ *n* (%)	97 (94–100)	97 (95–100)	97 (94–100)	0.231
Nbpm mmHg	76 (66–88)	76 (66–88)	74 (65–88)	0.353
*Laboratory parameters*				
cTnT ng/ml	0.09 (0.04–0.26)	0.08 (0.04–0.24)	0.11 (0.04–0.35)	0.003
HCT *n* (%)	30.8 (26.5–35.6)	30.7 (26.5–35.4)	31 (26.4–36)	0.060
Hb g/dL	9.9 (8.5–11.5)	9.9 (8.5–11.4)	9.9 (8.5–11.7)	0.081
PLT K/μL	190 (126–269)	194 (129–271)	178.5 (109.75–264.25)	0.013
RDW *n* (%)	15.6 (14.3–17.4)	15.5 (14.3–17.3)	15.8 (14.5–17.7)	0.002
RBC m/μL	3.35 (2.86–3.91)	3.36 (2.88–3.91)	3.325 (2.82–3.922)	0.711
WBC K/μL	13.2 (8.875–19.325)	13.1 (8.775–19.3)	13.5 (9.2–19.4)	0.028
Alb g/dL	2.7 (2.3–3.1)	2.8 (2.4–3.1)	2.6 (2.2–3.1)	<0.001
Glu mg/dL	138 (108–190)	137 (107–190)	144 (108–192)	0.397
Cl^−^ mEq/L	103 (99–108)	103 (99–108)	104 (99–109)	0.419
AG mEq/L	16 (13–19)	16 (13–19)	16 (14–19)	0.001
Ca^2+^ mg/dL	8.1 (7.5–8.6)	8.1 (7.5–8.6)	8 (7.5–8.6)	0.177
K^+^ mEq/L	4.2 (3.7–4.7)	4.2 (3.7–4.7)	4.3 (3.8–4.8)	0.032
Na^+^ mEq/L	138 (135–142)	138 (135–141.25)	138 (135–142)	0.113
PaCO_2_ mmHg	40 (34–48)	40 (34–48)	41 (34–50)	0.064
pH n	7.34 (7.27–7.4)	7.35 (7.28–7.41)	7.32 (7.24–7.39)	<0.001
PaO_2_ mmHg	68 (42–115)	68.5 (42–114)	68 (43–115.25)	0.966
INR n	1.4 (1.2–1.8)	1.4 (1.2–1.8)	1.5 (1.2–1.9)	0.515
PT sec	15.5 (13.4–19.8)	15.4 (13.3–19.6)	15.9 (13.7–20.8)	0.408
Lac mmol/L	1.9 (1.3–3.2)	1.9 (1.3–3)	2.2 (1.5–3.9)	<0.001
APTT sec	33.55 (28.9–42.7)	33.2 (28.8–41.625)	35 (29.1–47.8)	<0.001
ALT IU/L	29 (16–67)	29 (16–63)	31 (17–85)	0.003
AST IU/L	47 (26–106.25)	45 (25–96)	56 (29–149.25)	0.003
TB mg/dL	0.7 (0.4–1.3)	0.6 (0.4–1.2)	0.8 (0.4–1.9)	<0.001
Scr mg/dL	1.6 (1–2.7)	1.6 (1–2.7)	1.7 (1.1–2.8)	0.723
BUN mg/dL	35 (22–56)	34 (21–54)	38 (24–60)	<0.001
LDH IU/L	308 (228–463.25)	294 (219.75–422.25)	387 (264.75–609)	<0.001
SF ng/ml	549 (274.5–1,011)	513 (249–949)	715.5 (359–1,247)	<0.001
SI μg/dl	31 (20–50)	30 (20–49)	32 (18–56)	0.032
TIBC μg/dl	174 (134.75–215)	179 (142–221)	157 (114–194)	<0.001
TF mg/dl	133 (102–166)	136 (106–171)	121 (93–149.25)	<0.001
*Clinical severity*				
SOFA	8 (5–10)	7 (5–10)	9 (6–12)	<0.001
APS III	60 (47–75)	57 (46–72)	70 (55–87.25)	<0.001
SIRS	3 (3–4)	3 (3–4)	3 (3–4)	0.024
SAPS II	47 (38–56)	45 (36–54)	53 (43.75–63)	<0.001
OASIS	37 (31–43)	36 (30–42)	41 (35–47)	<0.001
GCS	15 (13–15)	15 (13–15)	15 (12–15)	<0.001
Charlson	7 (5–9)	7 (5–8)	7 (5–9)	<0.001
*Comorbidities*				
HTN				
	2,367 (70.45)	1813 (70.38)	554 (70.66)	0.915
Yes	993 (29.55)	763 (29.62)	230 (29.34)	
AKI				
	974 (28.99)	800 (31.06)	174 (22.19)	<0.001
Yes	2,386 (71.01)	1776 (68.94)	610 (77.81)	
T2DM				
	2025 (60.27)	1,534 (59.55)	491 (62.63)	0.134
Yes	1,335 (39.73)	1,042 (40.45)	293 (37.37)	
HLD				
	2,105 (62.65)	1,611 (62.54)	494 (63.01)	0.844
Yes	1,255 (37.35)	965 (37.46)	290 (36.99)	
CKD				
	2,212 (65.83)	1,696 (65.84)	516 (65.82)	1.000
Yes	1,148 (34.17)	880 (34.16)	268 (34.18)	
*Treatment*				
CRRT				
	2,825 (84.08)	2,230 (86.57)	595 (75.89)	<0.001
Yes	535 (15.92)	346 (13.43)	189 (24.11)	
Ventilation				
	363 (10.80)	284 (11.02)	79 (10.08)	0.494
Yes	2,997 (89.20)	2,292 (88.98)	705 (89.92)	
Ferrous preparation PO				
	3,061 (91.10)	2,322 (90.14)	739 (94.26)	0.001
Yes	299 (8.90)	254 (9.86)	45 (5.74)	
Ferrous preparation IV				
	3,148 (93.69)	2,384 (92.55)	764 (97.45)	<0.001
Yes	212 (6.31)	192 (7.45)	20 (2.55)	
Phenylephrine				
	1,837 (54.67)	1,493 (57.96)	344 (43.88)	<0.001
Yes	1,523 (45.33)	1,083 (42.04)	440 (56.12)	
Norepine				
	855 (25.45)	748 (29.04)	107 (13.65)	<0.001
Yes	2,505 (74.55)	1,828 (70.96)	677 (86.35)	
*Outcomes*				
SIMI (%)				
	2,728 (81.19)	2,123 (82.41)	605 (77.17)	0.001
Yes	632 (18.81)	453 (17.59)	179 (22.83)	

HR, heart rate; Nbpm, non-invasive blood pressure mean; RR, respiratory rate; SpO_2_, oxygen saturation; T, body temperature; CRRT, renal replacement therapy; PO, per os; IV, intravenous; AKI, acute kidney injury; CKD, chronic kidney disease; HLD, hyperlipidemia; HTN, hypertension; T2DM, type 2 diabetes mellitus; RBC, red blood cell count; WBC, white blood cell count; RDW, red cell distribution width; PLT, platelet count; Hb, hemoglobin; HCT, hematocrit; TB, total bilirubin; ALT, alanine aminotransferase; Glu, serum glucose; Alb, albumin; AST, aspartate aminotransferase; PT, prothrombin time; INR, international normalized ratio; APTT, activated partial thromboplastin time; Scr, serum creatinine; BUN, blood urea nitrogen; AG, anion gap; PO_2_, partial pressure of oxygen; PCO_2_, partial pressure of carbon dioxide; K^+^, serum potassium; Na^+^, serum sodium; Ca^2+^, serum calcium; Cl^−^, serum chloride; cTnT, cardiac troponin T; SI, serum iron; SF, ferritin; TF, transferrin; TIBC, total iron-binding capacity; LDH, lactate dehydrogenase; Lac, lactate; SOFA, Sequential Organ Failure Assessment; APS III, Acute Physiology Score III; SIRS, Systemic Inflammatory Response Syndrome; SAPS II, Simplified Acute Physiology Score II; OASIS, Oxford Acute Severity of Illness Score; GCS, Glasgow Coma Scale; Charlson, Charlson Comorbidity Index; SIMI, sepsis-induced myocardial injury.

### 3.2 Association between iron metabolism markers in sepsis patients

#### 3.2.1 Survival analysis

**Figure 2:**
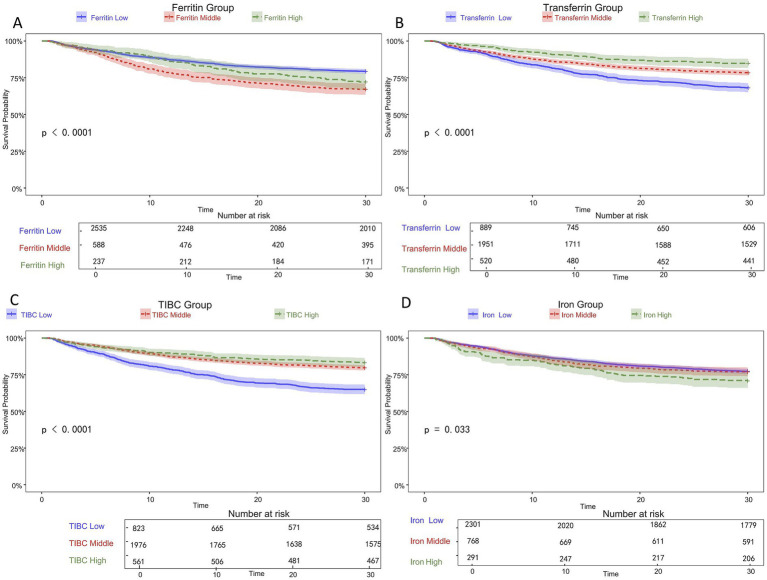
Kaplan–Meier survival curves for sepsis patients stratified by iron metabolism marker levels. **(A)** SF of 30-day survival analysis; **(B)** TF of 30-day survival analysis; **(C)** TIBC of 30-day survival analysis; **(D)** SI of 30-day survival analysis. The x-axis represents survival time, and the y-axis indicates the cumulative survival probability, SF, serum ferritin; TF, transferrin; TIBC, total iron-binding capacity; SI, serum iron.

The four iron metabolism markers were categorized into low, medium, and high groups using equal-width quantile binning for subsequent analysis. As shown in Figure 2, Kaplan–Meier survival analysis revealed that patients with medium ferritin levels, low transferrin levels, low TIBC (*p* < 0.001), and high serum iron levels (*p* = 0.033) exhibited the highest mortality.

#### 3.2.2 Correlation of the iron metabolism markers with outcome events

Subsequently, the association between iron metabolism markers and 30-day mortality was analyzed by constructing three Cox proportional hazards models (Table 3). As shown in Table 3, using Model 1 as the reference, in Model 3, ferritin (High: HR = 1.56, 95% CI: 1.195–2.038, *p* < 0.001) was associated with an increased risk, whereas transferrin (High: HR = 0.552, 95% CI: 0.425–0.718, *p* < 0.001) and total iron-binding capacity (TIBC; High: HR = 0.559, 95% CI: 0.435–0.717, *p* < 0.001) demonstrated protective effects. No significant difference was observed for serum iron levels.

**Table 3:** Cox proportional hazard ratios with 30-day mortality as the outcome event.

Variables	Model 1		Model 2		Model 3	
	HR (95% CI)	*P*	HR (95% CI)	*P*	HR (95% CI)	*P*
Ferritin						
Low	1.00 (Reference)		1.00 (Reference)		1.00 (Reference)	
Middle	1.712 (1.451–2.019)	< 0.001	1.746 (1.480–2.061)	< 0.001	1.512 (1.274–1.794)	< 0.001
High	1.365 (1.057–1.763)	< 0.017	1.525 (1.178–1.975)	0.001	1.560 (1.195–2.038)	0.001
*P* for trend	1.308 (1.180–1.450)	< 0.001	1.372 (1.236–1.524)	< 0.001	1.330 (1.190–1.486)	< 0.001
Transferrin						
Low	1.00 (Reference)		1.00 (Reference)		1.00 (Reference)	
Middle	0.645 (0.555–0.750)	< 0.001	0.644 (0.553–0.749)	< 0.001	0.743 (0.635–0.870)	< 0.001
High	0.434 (0.338–0.556)	< 0.001	0.428 (0.332–0.550)	< 0.001	0.552 (0.425–0.718)	< 0.001
*P* for trend	0.654 (0.584–0.731)	< 0.001	0.650 (0.581–0.728)	< 0.001	0.743 (0.660–0.837)	< 0.001
TIBC						
Low	1.00 (Reference)		1.00 (Reference)		1.00 (Reference)	
Middle	0.525 (0.451–0.611)	< 0.001	0.512 (0.439–0.597)	< 0.001	0.616 (0.525–0.724)	< 0.001
High	0.427 (0.339–0.539)	< 0.001	0.423 (0.334–0.536)	< 0.001	0.559 (0.435–0.717)	< 0.001
*P* for trend	0.608 (0.543–0.681)	< 0.001	0.600 (0.535–0.673)	< 0.001	0.703 (0.623–0.793)	< 0.001
Serum iron						
Low	1.00 (Reference)		1.00 (Reference)		1.00 (Reference)	
Middle	1.027 (0.866–1.218)	0.755	1.051 (0.884–1.249)	0.572	0.924 (0.773–1.103)	0.381
High	1.354 (1.077–1.703)	0.009	1.463 (1.162–1.843)	0.001	1.087 (0.855–1.382)	0.496
*P* for trend	1.123 (1.011–1.247)	0.03	1.162 (1.045–1.292)	0.005	1.008 (0.902–1.125)	0.893

HR, hazard ratio; CI, confidence interval; TIBC, total iron-binding capacity.

Model 1: Crude.

Model 2: Adjusted: age, weight, gender, race, signs (HR, T, RR, SpO_2_, Nbpm).

Model 3: Adjusted: age, weight, gender, race, vital signs (HR, T, RR, SpO_2_, Nbpm), clinical severity (SOFA, APS III, SAPS II, OASIS, GCS, SIRS, Charlson), laboratory parameters (RBC, RDW, WBC, PLT, HCT, Hb, Alb, Ca^2+^, Na^+^, K^+^, Cl^−^, Glu, AG, ALT, AST, TB, Scr, BUN, INR, PT, APTT, PaCO_2_, PaO_2_, pH, LDH, lactate, cTnT), comorbidities (HTN, AKI, CKD, T2DM, HLD), treatment (CRRT, ventilation, epinephrine, norepinephrine).

**Figure 3:**
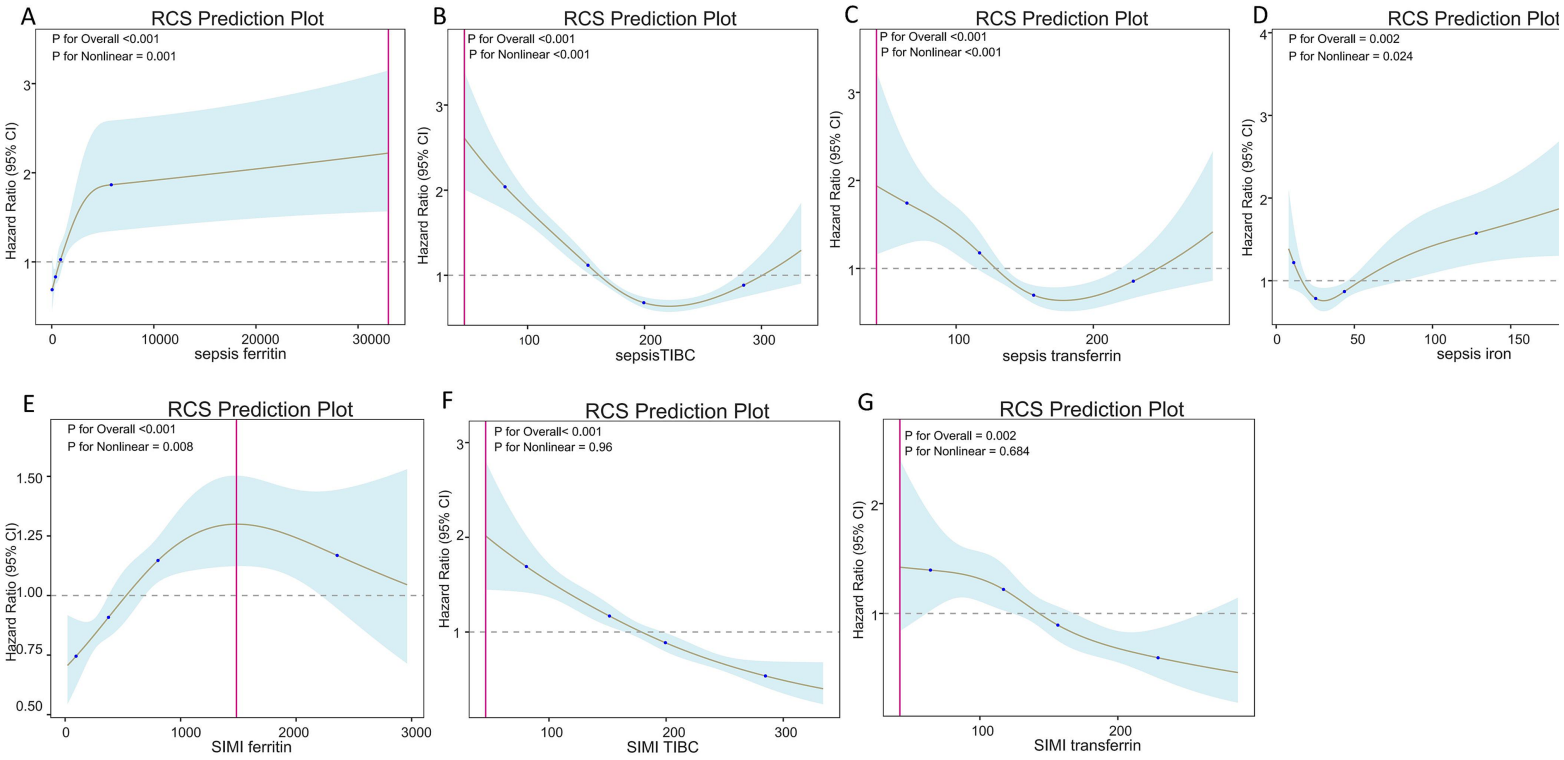
Restricted cubic spline analysis of association of iron metabolism markers with 30-day mortality and SIMI **(A)** SF with 30-day mortality; **(B)** TIBC with 30-day mortality; **(C)** TF with 30-day mortality; **(D)** SI with 30-day mortality; **(E)** SF with SIMI; **(F)** TIBC with SIMI; **(G)** TF with SIMI. SF, serum ferritin; TF, transferrin; TIBC, total iron-binding capacity; SI, serum iron; SIMI, sepsis-induced myocardial injury.

RCS analysis further revealed a nonlinear association between the four iron metabolism markers and 30-day mortality (Figures 3A–D, *p* < 0.05). Meanwhile, a nonlinear association was also observed between ferritin and SIMI (Figure 3E, *p* < 0.05), whereas linear associations were identified for TIBC and transferrin with SIMI (Figures 3F,G, *p* < 0.05). The results of the threshold effect analysis are presented in Supplementary Table S3.

### 3.3 Baseline characteristics after propensity score matching

Table 4 presents the baseline characteristics after matching. Variables included in the PSM encompassed basic vital signs, laboratory parameters, comorbidities, disease severity scores, and treatments. Matching improved the balance between variables, with absolute SMDs less than 0.10. The results indicated that patients in the intravenous iron group had a lower 30-day mortality rate. No significant benefit was observed in the oral iron group, and no association was found between iron administration and the occurrence of myocardial injury. Significant differences were noted in the incidence of complications, including AKI, CKD, CRRT, and HTN, between the oral and intravenous iron groups. These disparities may influence patient prognosis.

**Table 4:** Baseline characteristics after propensity score matching.

Variables	None	Ferrous preparation IV	SMD	None	Ferrous preparation PO	SMD
*N*	187	187		290	290	
*Demographic variables*						
Age years	70 (59–79)	70 (60–79)	0.030	74 (63.25–83)	74 (63.25–83)	0.035
Gender (%)						
Female	78 (41.71)	78 (41.71)	<0.001	121 (41.72)	122 (42.07)	0.007
Male	109 (58.29)	109 (58.29)		169 (58.28)	168 (57.93)	
Race (%)						
White	116 (62.03)	113 (60.43)	0.043	191 (65.86)	196 (67.59)	0.052
Black	38 (20.32)	38 (20.32)		59 (20.34)	53 (18.28)	
Other	33 (17.65)	36 (19.25)		40 (13.79)	41 (14.14)	
Weight lbs	79.7 (64.225–97)	75 (63.95–93.167)	0.024	79.65 (66.7–97.35)	78.4 (64.55–94.475)	0.111
*Vital signs*						
HR bpm	89 (78–105)	90 (77–104)	0.001	93 (77.25–109)	92 (80–108.75)	0.035
RR insp/min	20 (17–24)	20 (16–24.5)	0.018	20 (17–25)	20.5 (17–26)	0.052
T °F	98.2 (97.7–98.8)	98.1 (97.6–98.95)	0.105	98.4 (97.7–99.1)	98.2 (97.6–98.9)	0.059
SpO_2_ *n* (%)	98 (95–100)	97 (95–100)	0.046	97 (95–100)	97 (94–99)	0.017
Nbpm mmHg	74 (64–85.5)	75 (65.5–87.5)	0.077	75.5 (65–91)	75 (64–87)	0.098
*Laboratory parameters*						
cTnT ng/ml	0.13 (0.055–0.31)	0.16 (0.06–0.385)	0.124	0.1 (0.04–0.268)	0.09 (0.04–0.288)	0.005
HCT *n* (%)	28.6 (25.05–32.2)	27.9 (24.8–32.55)	0.001	29.2 (25.4–34.175)	29.35 (25.5–32.975)	0.047
Hb g/dL	8.9 (7.7–10.1)	8.9 (7.85–10.1)	0.037	9.3 (8.1–10.9)	9.3 (8.2–10.675)	0.060
PLT K/μL	191 (123–273.5)	195 (126–263)	0.002	209.5 (144.5–277.75)	204 (133.25–286)	0.011
RDW *n* (%)	17.2 (15.3–19.4)	16.7 (15.35–18.5)	0.134	15.9 (14.5–17.8)	15.95 (14.725–17.5)	0.032
RBC m/μL	3.15 (2.73–3.53)	3.01 (2.7–3.52)	0.005	3.255 (2.765–3.83)	3.195 (2.782–3.738)	0.074
WBC K/μL	11.8 (7.95–17.8)	13.3 (8.85–18.55)	0.088	13.25 (8.9–19.075)	13 (8.525–18.175)	0.008
Alb g/dL	2.7 (2.3–3.05)	2.7 (2.3–3.1)	0.014	2.7 (2.3–3.1)	2.7 (2.3–3.1)	0.002
Glu mg/dL	132 (101.5–190.5)	138 (107.5–194.5)	0.110	145 (106.25–203)	132 (104–188.75)	0.069
Cl^−^ mEq/L	99 (94–103)	98 (95–103)	0.053	103 (97–108)	102 (98–107.75)	0.022
AG mEq/L	17 (14.5–21)	17 (14–20)	0.071	16 (13–19)	15 (13–18)	0.056
Ca^2+^ mg/dL	8.3 (7.8–8.8)	8.3 (7.8–8.9)	0.007	8.1 (7.6–8.7)	8 (7.6–8.6)	0.053
K^+^ mEq/L	4.5 (3.9–5.1)	4.4 (3.8–5)	0.113	4.2 (3.8–4.8)	4.2 (3.7–4.7)	0.008
Na^+^ mEq/L	136 (132–139)	137 (132–140)	0.073	138 (134–141)	137 (134–141)	0.026
PaCO_2_ mmHg	42 (36–52)	43 (37–50.5)	0.065	41 (34–47)	40 (34–48)	0.079
pH n	7.34 (7.27–7.39)	7.35 (7.28–7.42)	0.123	7.36 (7.29–7.41)	7.35 (7.3–7.41)	0.040
PaO_2_ mmHg	51 (38–102)	68 (41–127.5)	0.177	65.5 (40–108.75)	70 (44–104)	0.028
INR n	1.5 (1.25–1.9)	1.4 (1.2–1.9)	0.127	1.4 (1.2–1.8)	1.4 (1.2–1.8)	0.057
PT sec	16.4 (13.7–20.75)	15.2 (13.1–20.9)	0.142	15 (13.3–19.8)	15.05 (13.2–19.275)	0.039
Lac mmol/L	1.7 (1.15–2.85)	1.6 (1.1–2.4)	0.058	1.9 (1.3–2.975)	1.7 (1.3–2.875)	0.050
APTT sec	33.7 (29.55–48.65)	34.4 (30–45.4)	0.058	34.8 (29–45.075)	33.4 (29.425–41.675)	0.097
ALT IU/L	29 (17.5–56.5)	24 (14–56)	0.056	25 (15–54)	28.5 (14–51)	0.074
AST IU/L	42 (25–77)	39 (23–91)	0.077	34 (22–88)	40.5 (23–85)	0.016
TB mg/dL	0.6 (0.3–1.2)	0.6 (0.4–1.15)	0.005	0.6 (0.4–1)	0.5 (0.3–1)	0.049
Scr mg/dL	2.9 (1.4–4.9)	3.4 (2.05–5.4)	<0.001	1.8 (1.125–3)	1.7 (1.1–2.9)	0.062
BUN mg/dL	43 (28.5–72)	48 (25–73)	0.017	36 (21–56)	38.5 (22–57.75)	0.011
LDH IU/L	314 (233–465.5)	281 (214.5–415)	0.002	291.5 (227–391.25)	288.5 (219–406.25)	0.009
SF ng/ml	701 (329.5–1,313)	652 (313.5–1181.5)	0.025	460 (229.5–944.75)	518 (266.75–940.25)	0.023
SI μg/dl	35 (19.5–58)	33 (21–51.5)	0.057	27 (17–43.75)	27 (17.25–44)	0.053
TIBC μg/dl	178 (143–215)	174 (137–219.5)	0.068	179 (139–228)	176 (142.25–223.5)	0.072
TF mg/dl	132 (101.5–167)	134 (99–169.5)	0.025	144 (112–179.75)	134 (106.25–174)	0.140
*Clinical severity*						
SOFA	9 (6–11)	8 (6–11)	0.054	7 (5–10)	7 (5–10)	0.081
APSIII	64 (50–76)	62 (50–76)	0.034	60 (48–75)	59 (48–73)	0.017
SIRS	3 (2–4)	3 (2–3)	0.031	3 (2–3)	3 (2–4)	0.025
SAPSII	47 (36–57)	46 (36.5–54)	0.089	46 (37–56)	45 (36–55)	0.038
OASIS	37 (29.5–43)	37 (29–43)	0.020	37 (31–43)	36 (30–42)	0.070
GCS	15 (13–15)	15 (13–15)	0.081	15 (13–15)	15 (13–15)	0.034
Charlson	8 (6–10)	8 (6–9)	0.080	7 (5–9)	7 (5–9)	0.039
*Comorbidities*						
HTN						
	176 (94.12)	171 (91.44)	0.103	206 (71.03)	206 (71.03)	<0.001
Yes	11 (5.88)	16 (8.56)		84 (28.97)	84 (28.97)	
AKI						
	73 (39.04)	80 (42.78)	0.076	87 (30.00)	91 (31.38)	0.030
Yes	114 (60.96)	107 (57.22)		203 (70.00)	199 (68.62)	
T2DM						
	92 (49.20)	89 (47.59)	0.032	139 (47.93)	158 (54.48)	0.131
Yes	95 (50.80)	98 (52.41)		151 (52.07)	132 (45.52)	
HLD						
	115 (61.50)	114 (60.96)	0.011	164 (56.55)	168 (57.93)	0.028
Yes	72 (38.50)	73 (39.04)		126 (43.45)	122 (42.07)	
CKD						
	87 (46.52)	90 (48.13)	0.032	172 (59.31)	170 (58.62)	0.014
Yes	100 (53.48)	97 (51.87)		118 (40.69)	120 (41.38)	
*Treatment*						
CRRT						
	121 (64.71)	118 (63.10)	0.033	253 (87.24)	252 (86.90)	0.010
Yes	66 (35.29)	69 (36.90)		37 (12.76)	38 (13.10)	
Ventilation						
	18 (9.63)	17 (9.09)	0.018	34 (11.72)	30 (10.34)	0.044
Yes	169 (90.37)	170 (90.91)		256 (88.28)	260 (89.66)	
*Outcomes*			*P*			*P*
30-days mortality (%)						
	135 (72.19)	168 (89.84)	<0.001	232 (80.00)	247 (85.17)	0.125
Yes	52 (27.81)	19 (10.16)		58 (20.00)	43 (14.83)	
SIMI (%)						
	151 (80.75)	144 (77.01)	0.447	244 (84.14)	248 (85.52)	0.728
Yes	36 (19.25)	43 (22.99)		46 (15.86)	42 (14.48)	

HR, heart rate; Nbpm, non-invasive blood pressure mean; RR, respiratory rate; SpO_2_, oxygen saturation; T, body temperature; CRRT, renal replacement therapy; PO, per os; IV, intravenous; AKI, acute kidney injury; CKD, chronic kidney disease; HLD, hyperlipidemia; HTN, hypertension; T2DM, type 2 diabetes mellitus; RBC, red blood cell count; WBC, white blood cell count; RDW, red cell distribution width; PLT, platelet count; Hb, hemoglobin; HCT, hematocrit; TB, total bilirubin; ALT, alanine aminotransferase; Glu, serum glucose; Alb, albumin; AST, aspartate aminotransferase; PT, prothrombin time; INR, international normalized ratio; APTT, activated partial thromboplastin time; Scr, serum creatinine; BUN, blood urea nitrogen; AG, anion gap; PO_2_, partial pressure of oxygen; PCO_2_, partial pressure of carbon dioxide; K^+^, serum potassium; Na^+^, serum sodium; Ca^2+^, serum calcium; Cl^−^, serum chloride; cTnT, cardiac troponin T; SI, serum iron; SF, ferritin; TF, transferrin; TIBC, total iron-binding capacity; LDH, lactate dehydrogenase; Lac, lactate; SOFA, Sequential Organ Failure Assessment; APS III, Acute Physiology Score III; SIRS, Systemic Inflammatory Response Syndrome; SAPS II, Simplified Acute Physiology Score II; OASIS, Oxford Acute Severity of Illness Score; GCS, Glasgow Coma Scale; Charlson, Charlson Comorbidity Index; SIMI, sepsis-induced myocardial injury.

### 3.4 Survival analysis and sensitivity analysis of iron supplementation regimen

**Figure 4:**
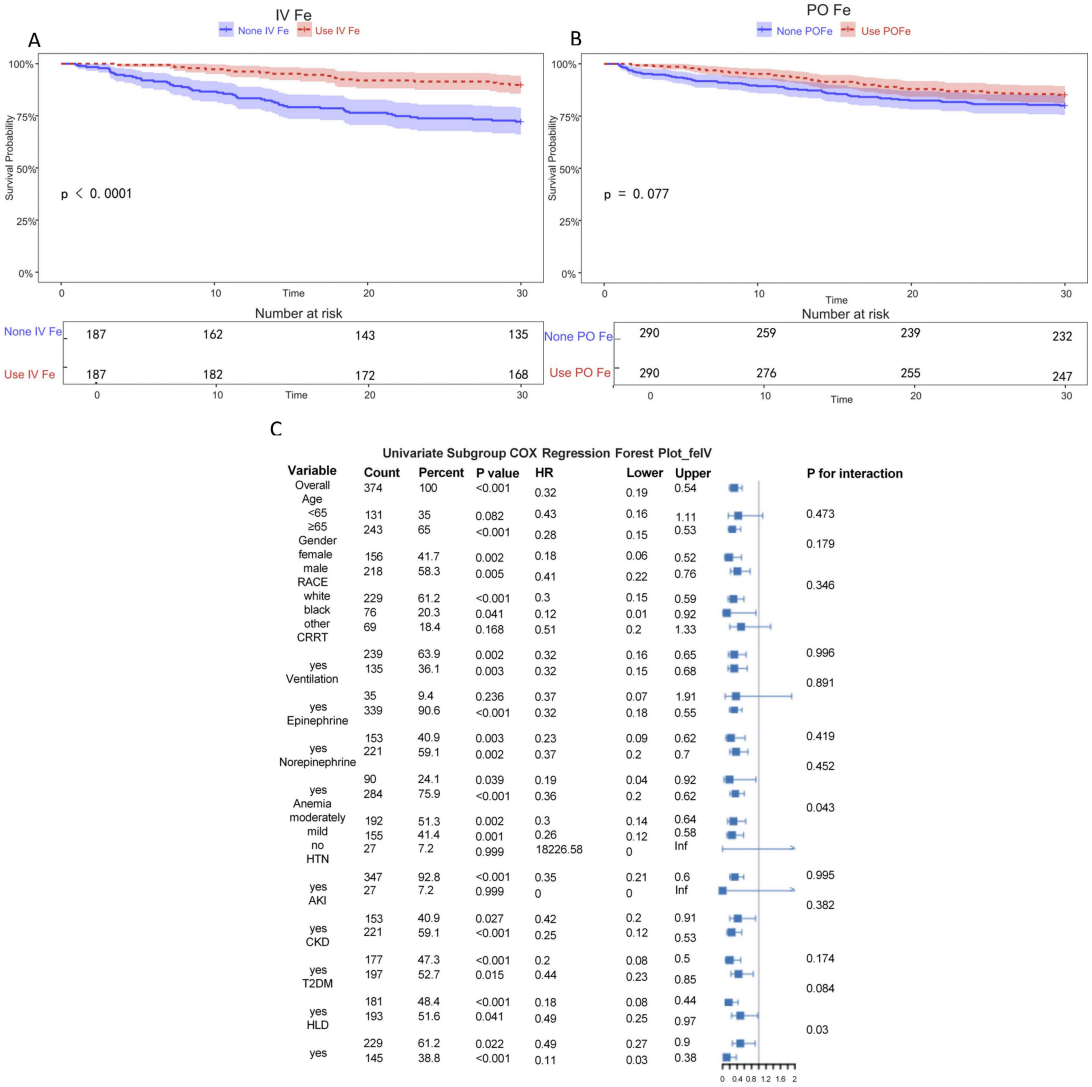
**(A)** Kaplan–Meier curves for 30-day mortality according to ferrous preparation IV; **(B)** Kaplan–Meier curves for 30-day mortality according to ferrous preparation PO; **(C)** The forest plot presents the results of the stratified analysis of the association between ferrous preparation IV and 30-day survival. CRRT, renal replacement therapy; HTN, hypertension; AKI, acute kidney injury; CKD, chronic kidney disease; T2DM, type 2 diabetes mellitus; HLD, hyperlipidemia; PO, per os; IV, intravenous.

After PSM, the intravenous iron group exhibited a significantly lower 30-day mortality rate (10.16% vs. 27.81%, *p* < 0.001). However, no significant improvement in prognosis was observed in the oral iron group, and no significant change in the incidence of myocardial injury was observed in either group. Kaplan–Meier survival analysis showed that after PSM, the 30-day mortality rate was significantly lower in the intravenous iron group, whereas no significant difference was observed in the oral iron group (Figures 4A,B). Cox proportional hazards regression analysis demonstrated that intravenous iron therapy was independently associated with a reduced risk of 30-day all-cause mortality across all models. In the unadjusted model, the HR was 0.322 (95% CI: 0.190–0.544; *p* < 0.001). In the partially adjusted model, the HR was 0.313 (95% CI: 0.185–0.531; *p* < 0.001). In the fully adjusted model, the HR remained significant at 0.251 (95% CI: 0.128–0.493; *p* = 0.001) (Table 5). Subgroup analyses were conducted based on age, sex, race, comorbidities (anemia, HTN, AKI, CKD, T2DM, and HLD), baseline treatments (CRRT, mechanical ventilation), and baseline medications (epinephrine, norepinephrine). Figure 4C presents the results of the subgroup analysis for 30-day all-cause mortality in the matched cohort. Notably, although no significant benefit of intravenous iron therapy was observed in subgroups of patients aged < 65 years, those without mechanical ventilation, those without anemia, and those without hypertension, a protective effect was consistently observed in the vast majority of subgroups. Furthermore, significant interactions were identified between the effectiveness of intravenous iron and the status of hyperlipidemia and anemia (interaction *p* < 0.05).

**Table 5:** Survival results of the group before and after propensity score matching.

Variables	Model 1		Model 2		Model 3	
	HR (95% CI)	*P*	HR (95% CI)	*P*	HR (95% CI)	*P*
After PSM						
Ferrous preparation IV	0.322 (0.19–0.544)	**< 0.001**	0.313 (0.185–0.531)	**< 0.001**	0.251 (0.128–0.493)	**< 0.001**
Before PSM						
Ferrous preparation IV	0.35 (0.224–0.545)	**< 0.001**	0.413 (0.265–0.646)	**< 0.001**	0.343 (0.216–0.544)	**< 0.001**

HR, hazard ratio; CI, confidence interval; IV, intravenous. Bold values mean PSM, propensity score matching.

Model 1: Crude.

Model 2: Adjusted: age, weight, gender, race, signs (HR, T, RR, SpO_2_, Nbpm).

Model 3: Adjusted: age, weight, gender, race, vital signs (HR, T, RR, SpO_2_, Nbpm), clinical severity (SOFA, APS III, SAPS II, OASIS, GCS, SIRS, Charlson), laboratory parameters (RBC, RDW, WBC, PLT, HCT, Hb, Alb, Ca^2+^, Na^+^, K^+^, Cl^−^, Glu, AG, ALT, AST, TB, Scr, BUN, INR, PT, APTT, PaCO_2_, PaO_2_, pH, LDH, lactate, cTnT), comorbidities (HTN, AKI, CKD, T2DM, HLD), treatment (CRRT, ventilation, epinephrine, norepinephrine).

## 4 Discussion

This study analyzed data from 3,360 patients with sepsis admitted to the ICU to investigate the associations among iron metabolism biomarkers, iron supplementation therapy, 30-day all-cause mortality, and the incidence of SIMI. Initially, a nonlinear relationship was observed between iron metabolism biomarkers and the SIMI. Subsequent to PSM, a reduction in 30-day mortality was noted in patients subjected to intravenous iron therapy, whereas no difference in 30-day mortality was observed in the group receiving oral iron therapy. Furthermore, no difference was detected in the incidence of SIMI between the iron supplementation group and non-iron supplementation group. These findings suggest that individualized assessment of iron status and judicious use of intravenous iron supplementation may represent a promising strategy for optimizing the management and prognosis of critically ill patients with sepsis.

Previous studies on the association between iron status and infection risk have suggested a nonlinear relationship (21, 22), indicating that both iron deficiency and iron overload are associated with an increased risk of infection (23–26). This finding is consistent with the results of the present study. Furthermore, we identified a nonlinear relationship between iron status and SIMI and determined the inflection point through threshold effect analysis.

Whether iron supplementation is necessary for patients with sepsis remains controversial. Animal experiments have demonstrated that exogenous iron administration can induce systemic oxidative stress in mice, leading to increased mortality (13, 27). Even anemic mice may experience exacerbated infections following iron supplementation (28). However, other studies have reported that intravenous iron administration does not increase mortality in mice and appears to have minimal adverse effects (15). Clinical studies have found that intramuscular iron supplementation may increase the occurrence rate of neonatal sepsis (29), whereas intravenous iron supplementation appears to be safer and more effective (16) and does not exacerbate infection symptoms in dialysis patients (30). These findings are consistent with the observation in the present study that the intravenous iron supplementation group had a lower mortality rate. Previous studies have overlooked the nonlinear relationship between iron status and sepsis. Failure to dynamically monitor iron metabolism status during experiments and the indiscriminate administration of iron supplements may account for the conflicting results observed across studies.

The classical pathways of sepsis-induced myocardial injury are closely associated with iron metabolism, as primarily reflected in the following aspects: inflammatory cytokines, such as interleukin-6 (IL-6), upregulate the expression of hepcidin, thereby inhibiting intestinal iron absorption and macrophage-mediated iron release. This leads to decreased plasma iron levels and increased intracellular iron accumulation, creating conditions conducive to ferroptosis. Tumor necrosis factor-alpha (TNF-*α*) can induce ferritinophagy, elevating intracellular levels of ferrous iron (Fe^2+^), which promotes lipid peroxidation and ferroptosis (31). Under conditions of iron metabolic disorders, mitochondrial iron overload exacerbates the production of reactive oxygen species (ROS). Through the Fenton reaction, hydroxyl radicals are generated, compromising mitochondrial membrane integrity and activating ferroptosis pathways (32). In sepsis, the expression of nuclear receptor coactivator 4 (NCOA4) is upregulated, promoting the degradation of ferritin and releasing substantial amounts of Fe^2+^. This results in intracellular iron accumulation, activation of lipid peroxidation, and subsequent triggering of ferroptosis (33). Concurrently, inflammation and oxidative stress reduce the activity of glutathione peroxidase 4 (GPX4), impairing the effective clearance of lipid peroxides. Consequently, lipid ROS accumulate, cellular antioxidant capacity declines, and ferroptosis occurs (34).

This study also has several limitations. First, as a retrospective observational study, it may be subject to inherent biases and unmeasured confounding factors, potentially leading to biased results. Second, the database contains a significant number of missing values for indicators such as height, inflammatory markers, and lipid profiles. These variables were not included in the multivariate Cox regression analysis, which may affect the independent predictive validity of iron metabolism markers. Simultaneously, multiple biomarkers and clinical endpoints were assessed in the present study, which may elevate the risk of type I error. Therefore, the reported associations should be interpreted with caution. Third, this study only analyzed baseline iron metabolism indicators and did not explore their dynamic changes over time. Fourth, despite the use of PSM and multivariate analysis, the findings may still be influenced by residual bias and unmeasured confounding variables. As potential systematic differences in patients receiving iron therapy might have been overlooked during PSM, the association between intravenous iron therapy and reduced mortality may be somewhat overestimated. Furthermore, we did not investigate other potential side effects besides myocardial injury; the safety of iron supplementation requires further study. Fifth, although many studies define SIMI based on elevated cardiac injury markers, some studies also incorporate indicators such as ejection fraction to further assess “septic myocardial injury.” Limited by the database, we could not obtain echocardiographic information for these patients, which may have potentially excluded a small subset of patients presenting solely with systolic or diastolic dysfunction. Therefore, future prospective, multicenter studies are needed to further validate the optimal dosage, timing, and regimen of iron therapy, and deeply explore the specific molecular mechanisms linking iron metabolism disorders and sepsis-induced myocardial injury, providing a more solid evidence-based foundation for the precise treatment of sepsis.

## 5 Conclusion

In patients with sepsis, ferritin, transferrin, and TIBC were significantly associated with 30-day mortality and SIMI risk, suggesting that iron metabolism indicators possess certain prognostic stratification value. Meanwhile, intravenous iron supplementation was associated with a lower risk of 30-day mortality, and no increased risk of myocardial injury was observed; however, this association still requires further validation by prospective studies.

## Data Availability

The raw data supporting the conclusions of this article will be made available by the authors, without undue reservation.
