# Supplementary material for: The associations among iron metabolism markers, iron supplementation regimen, and sepsis-induced myocardial injury: a retrospective study
Source: Front Nutr. 2026 Jul 15;13:1858343. doi: 10.3389/fnut.2026.1858343 (PMC13414743; doi:10.3389/fnut.2026.1858343)
Supplement: Supplementary file 1 [file Data_Sheet_1.PDF]

Supplementary materials

Table S1. Disease codes included in the study..2

Table S2. Clinical outcomes of iron metabolism markers..2

Table S3. Threshold effect analysis of outcome.. 2-3

Table S1. Disease codes included in the study

MIMIC-IV Sepsis	ICD9	99591, 99592
	ICD10	A021, A327, A400, A401, A403, A408, A409, A4101, A4102, A411, A412, A413, A414, A4150, A4151, A4152, A4153, A4159, A4181, A4189, A419, A427, A5486, B377, R6520, R6521

Table S2. Clinical outcomes of iron metabolism markers

Variables	30-days mortality		*P*	SIMI		*P*
SF ng/ml	513 (5.4-3054)	715.5 (5.4-3032)	<0.001	532.5 (5.4-3054)	646 (21-3053)	0.027
SI μg/dl	30 (5-116)	32 (5-116)	0.032	31 (5-116)	31 (5-116)	0.361
TIBC μg/dl	179 (33-335)	157 (38-335)	<0.001	178 (33-335)	159 (38-333)	<0.001
TF mg/dl	136 (25-258)	121 (25-263)	<0.001	134 (25-263)	127 (29-256)	0.001

SF: serum ferritin, TF: transferrin, TIBC: total iron-binding capacity, SI: serum iron, SIMI: sepsis-induced myocardial injury.

Table S3. Threshold effect analysis of outcome

Variables	Outcome	HR (95% CI)	*P*
Sepsis SF ng/ml	Model1 Line Effect	1.000 (0.999-1.000)	0.239
	Model2 Threshold		
	<2605	1.108 (1.034-1.182)	<0.001
	>2605	1.046 (1.023-1.069)	0.015
	P for likelihood ratio test		<0.001
Sepsis SI μg/dl	Model1 Line Effect	1.001 (0.998-1.004)	0.268
	Model2 Threshold		
	<22	0.945 (0.903-0.989)	0.016
	>22	1.122 (1.065-1.179)	0.001

	P for likelihood ratio test		0.018
Sepsis TIBC μg/dl	Model1 Line Effect	1.001 (0.998-1.004)	0.268
	Model2 Threshold		
	<233	0.887 (0.798-0.976)	<0.001
	>233	1.162 (1.079-1.245)	<0.001
	P for likelihood ratio test		<0.001
Sepsis TF mg/dl	Model1 Line Effect	1.000 (0.993-1.000)	0.498
	Model2 Threshold		
	<179	0.908 (0.893-0.923)	<0.001
	>179	1.119 (1.081-1.157)	<0.001
	P for likelihood ratio test		<0.001
SIMI SF ng/ml	Model1 Line Effect	1.000 (1.000-1.000)	0.524
	Model2 Threshold		
	<1494	1.237 (1.109-1.364)	0.021
	>1494	0.937 (0.905-0.969)	0.011
	P for likelihood ratio test		0.038

SIMI: sepsis-induced myocardial injury, SF: serum ferritin, TF: transferrin, TIBC: total iron-binding capacity, SI: serum iron, HR: hazard ratio.
